# A New Approach for Noise Suppression in Cochlear Implants: A Single-Channel Noise Reduction Algorithm^1^

**DOI:** 10.3389/fnins.2020.00301

**Published:** 2020-04-21

**Authors:** Huali Zhou, Ningyuan Wang, Nengheng Zheng, Guangzheng Yu, Qinglin Meng

**Affiliations:** ^1^Acoustics Lab, School of Physics and Optoelectronics, South China University of Technology, Guangzhou, China; ^2^Nurotron Biotechnology Inc., Hangzhou, China; ^3^The Guangdong Key Laboratory of Intelligent Information Processing, College of Electronics and Information Engineering, Shenzhen University, Shenzhen, China

**Keywords:** cochlear implant, noise reduction, cocktail party problem, monaural, speech in noise, intelligibility, Nurotron, eVoice

## Abstract

The cochlea “translates” the in-air vibrational acoustic “language” into the spikes of neural “language” that are then transmitted to the brain for auditory understanding and/or perception. During this intracochlear “translation” process, high resolution in time–frequency–intensity domains guarantees the high quality of the input neural information for the brain, which is vital for our outstanding hearing abilities. However, cochlear implants (CIs) have coarse artificial coding and interfaces, and CI users experience more challenges in common acoustic environments than their normal-hearing (NH) peers. Noise from sound sources that a listener has no interest in may be neglected by NH listeners, but they may distract a CI user. We discuss the CI noise-suppression techniques and introduce noise management for a new implant system. The monaural signal-to-noise ratio estimation-based noise suppression algorithm “eVoice,” which is incorporated in the processors of Nurotron^®^ Enduro^TM^, was evaluated in two speech perception experiments. The results show that speech intelligibility in stationary speech-shaped noise can be significantly improved with eVoice. Similar results have been observed in other CI devices with single-channel noise reduction techniques. Specifically, the mean speech reception threshold decrease in the present study was 2.2 dB. The Nurotron society already has more than 10,000 users, and eVoice is a start for noise management in the new system. Future steps on non-stationary-noise suppression, spatial-source separation, bilateral hearing, microphone configuration, and environment specification are warranted. The existing evidence, including our research, suggests that noise-suppression techniques should be applied in CI systems. The artificial hearing of CI listeners requires more advanced signal processing techniques to reduce brain effort and increase intelligibility in noisy settings.

## Introduction

The cochlear implant (CI) is one of the most successful prostheses ever developed and aims to rehabilitate hearing by transmitting acoustic information into the brains of people with severe to profound hearing impairment by electrically stimulating auditory nerve fibers (Shannon, 2014). The artificial electric hearing provided by current CIs is useful for speech communication but is still far from satisfactory compared with normal hearing (NH), especially in the aspect of speech-in-noise recognition.

The noise issue is a common complaint of CI users (e.g., Ren et al., 2018). Because of variability associated with implant surgery time, hearing history, rehabilitation and training, surgical conditions, devices and signal processing, and so on, large differences in hearing abilities have always been reported within any group of CI users. These reasons behind the CI-NH gap and intersubject CI variance may be classified into “top-down” and “bottom-up” types (Moberly and Reed, 2019; Tamati et al., 2019).

From a practical standpoint, knowledge about “top-down” memory and cognition is useful for rehabilitation and making surgical decisions (Kral et al., 2019), whereas the relationship between speech performance and the “bottom-up” signal processing functions—especially those on the electrode interface—determines the engineering approaches used in current CI systems (Wilson et al., 1991; Loizou, 1999, 2006; Rubinstein, 2004; Zeng, 2004; Zeng et al., 2008; Wouters et al., 2015; Nogueira et al., 2018). Although the “top-down” approach has been suggested to be incorporated into CI systems to form an adaptive closed-loop neural prothesis (Mc Laughlin et al., 2012), we only introduce “bottom-up”–related techniques that might be useful for CI users to tackle the problem of noise masking, as discussed below.

How to send more useful information upward? Sound pressure waveforms are decomposed by healthy cochleae into fine temporal-spectral “auditory images”. CIs attempt to capture and deliver the same images but, unfortunately, in a coarse way. Theories in grouping, scene analysis, unmasking, and attention have demonstrated the significance of precise coding of acoustic cues including pitch or resolved harmonics, common onset, and spatial cues. For most CI systems, only temporal envelopes from a limited number of channels can be transferred to the nerve, and current interactions between channels are a key limitation of the multichannel CI framework.

Several research directions have been explored to improve the CI recognition performance of speech in noise by updating the technology of contemporary multichannel devices: (1) stimulating auditory nerves in novel physical ways such as optical stimulation (Jeschke and Moser, 2015) and penetrating nerve stimulation (Middlebrooks and Snyder, 2007); (2) developing intracochlear electrode arrays with different lengths, electrode shapes, and mechanical characteristics (Dhanasingh and Jolly, 2017; Rebscher et al., 2018; Xu et al., 2018); (3) steering and focusing the current spread by simultaneously activating multiple electrodes (Berenstein et al., 2008; Bonham and Litvak, 2008); (4) refining the strategies in the temporal domain by introducing harmonics (Li et al., 2012), timing of zero crossings (Zierhofer, 2003) or peaks (Van Hoesel, 2007), and slowly varying temporal fine structures (Nie et al., 2005; Meng et al., 2016); and (5) enhancing speech or suppressing noise before or within the core signal processing strategies. The first and second directions are developed from the perspective of neurophysiology; the third is mainly based on psychophysical tests; the fourth uses a combination of signal processing and psychophysics, and the fifth mainly concentrates on signal processing. All of these aspects are worth further investigation.

In the last two decades, the fifth approach of enhancing speech or suppressing noise before or within the core signal processing strategies has become a hot topic in academic and industrial research. Noise reduction and speech enhancement are two sides of the same coin, and the goal is to improve intelligibility or quality of speech in noise, in most cases with a signal-to-noise ratio (SNR) enhancement signal processing system. Some noise reduction techniques in telecommunications and hearing aids have been used to process noisy speech signals, and then the processed signals are presented through loudspeakers to CI users (e.g., classic single-channel spectral subtraction) (Yang and Fu, 2005) for feasibility verification. Now there are more sophisticated single-channel noise-reduction algorithms (NRAs) (Chen et al., 2015), directional microphone, or multimicrophone-based beamformers of hearing aids (Chung et al., 2004; Buechner et al., 2014), and more recently deep neural network–based algorithms (Lai et al., 2018; Goehring et al., 2019) that have been tried with CI listeners. Another line of research is to specifically optimize algorithm parameters with a consideration of the differences between CI and NH listeners. The parameters are generally related to the noise estimation or gain function for noise reduction (Hu et al., 2007; Kasturi and Loizou, 2007; Mauger et al., 2012a, b; Wang and Hansen, 2018). All these studies demonstrated significant improvements, which can be explained by the higher SNR yielded by the techniques before or within the CI core strategies.

In the newest versions of CI processors from current commercial companies such as Cochlear^®^ (Hersbach et al., 2012), Advanced Bionics^®^ (Buechner et al., 2010), and MED-EL^®^ (Hagen et al., 2019), one or multiple algorithms of SNR-based monaural noise reduction and spatial cue-based directional microphone or multimicrophone beamformers have been implemented and evaluated. Multimicrophone beamformers significantly improve speech intelligibility for CI recipients in noise. However, it is based on the assumption that target speech and noise sources are spatially separated. Thus, single-microphone NRAs in CI systems are still worthy of attention to improve speech perception in noise, especially in scenarios when the target speech and noise sources are not spatially separated.

Some single-microphone NRAs that are already implemented in commercial CI products have been reported in the literature. ClearVoice is a monaural NRA implemented with the HiRes 120 speech processing strategy (Buechner et al., 2010; Holden et al., 2013). It first estimates noise by assuming that speech energy amplitude changes frequently and background noise energy is less modulated. Then, gain is reduced for channels identified as having mainly noise energy. The noise estimation works at a time window of 1.3 s, which is the activation time of this algorithm. Experiments showed that ClearVoice can improve speech intelligibility in stationary noise (Buechner et al., 2010; Kam et al., 2012). Another monaural NRA is implemented with the ACE (advanced combination encoder) strategy in Nucleus devices. It uses a minimum statistics algorithm with an optimal smoothing method for noise estimation (Martin, 2001) and an *a priori* SNR estimate (McAulay and Malpass, 1980) in conjunction with a modified Wiener gain function (Loizou, 2007). It was reported to significantly improve hearing in stationary noise (Dawson et al., 2011).

We introduce a recently developed single-channel estimated-SNR–based NRA, termed “eVoice,” which has been implemented in the second-generation research processor Enduro^TM^ of Nurotron. Nurotron, a young company based in Irvine, CA, United States, and Hangzhou, Zhejiang, China, currently has more than 10,000 patients implanted. The Nurotron system has 24 electrode channels, and its users’ speech performance in quiet and postsurgery development status are comparable with previous data from other brands (Zeng et al., 2015; Gao et al., 2016). The noise estimation in eVoice is processed on a frame-by-frame basis, which is using a relatively short time window. It is based on classical signal processing algorithms and is not the first CI device to use this kind of approach. The aims of this study include reporting the intelligibility experiment results for eVoice and rethinking noise management of a new CI system, which in this case is the Nurotron system.

## eVoice of Nurotron: a Single-Channel NRA

**FIGURE 1:**
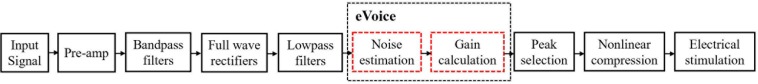
Block diagram of the APS strategy (black) and eVoice (red).

The default core strategy of Nurotron is the advanced peak selection (APS) strategy, which is similar to an “*n*-of-*m*” strategy (Zeng et al., 2015). The APS strategy is based on a short-time Fourier transform (STFT) and typically selects eight maxima (an automatic process defined in the coding strategy) for stimulation in each frame (Ping et al., 2017). A block diagram of the APS strategy and eVoice is shown in Figure 1. In APS, acoustic input signal is first preamplified, followed by bandpass filtering (the band number *m* typically equals the active electrode number, i.e., *m* = 24 in Nurotron devices) and envelope calculation. Then, in peak selection, *n* bands with the largest amplitude are selected for further non-linear compression and electrical stimulation (typically, *n* = 8 in Nurotron devices). The eVoice is an envelope-based noise reduction method implemented between envelope calculation and peak selection. It consists of two steps: noise estimation and gain calculation (Wang et al., 2017).

### Noise Estimation

The noise estimation algorithm is based on an improved minima-controlled recursive averaging (MCRA-2) algorithm (Rangachari and Loizou, 2006). Noise power in each channel is estimated on a frame-by-frame basis instead of a time window that includes several frames to reduce response time.

Suppose that the noise is additive, then in the time domain, the input signal *y*(*n*) can be denoted as

(1) y⁢(n)=x⁢(n)+d⁢(n)

where *x*(*n*) is the clean speech signal and *d*(*n*) is the additive noise signal. We use *Y*(λ, *k*), i.e., the STFT of *y*(*n*), to represent the summation magnitude of channel *k* in frame λ in the frequency domain. The power spectrum of the noisy signal can be smoothed and updated on a frame-by-frame basis using the recursion below:

(2) $$P\,\left(\lambda ,k\right)=\eta \,P\,\left(\lambda -1,k\right)+\left(1-\eta \right)\,{\left|Y\,\left(\lambda ,k\right)\right|}^{2}$$

where η is a smoothing factor. Then, the local minimum of the power spectrum in each channel can be tracked as follows:

(3) $$P_{m\,i\,n}\,\left(\lambda ,k\right)=\left\{ \begin{array}{cc} P\left(\lambda ,k\right)\;,P_{m\,i\,n}\left(\lambda -1,k\right)\geq P\left(\lambda ,k\right) & \\ \gamma \,P_{m\,i\,n}\,\left(\lambda -1,k\right)+\frac{1-\gamma }{1-\beta }\,\left(P\,\left(\lambda ,k\right)-\beta \,P\,\left(\lambda -1,k\right)\right), & \\ P_{m\,i\,n}\,\left(\lambda -1,k\right)<P\,\left(\lambda ,k\right) & \end{array}\right.$$

where *P*_*min*_(λ,*k*) is the local minimum of the noisy speech power spectrum, and β and γ are constant parameters. The ratio of noisy speech power spectrum to its local minimum can be calculated as follows:

(4) $$S_{r}\,\left(\lambda ,k\right)=\frac{P\,\left(\lambda ,k\right)}{P_{m\,i\,n}\,\left(\lambda ,k\right)}$$

This ratio is compared against a threshold *T*(λ,*k*) to determine the speech-presence probability *I*(λ,*k*) using the criterion below:

(5) $$I\,\left(\lambda ,k\right)=\left\{ \begin{array}{c} 1,S_{r}\,\left(\lambda ,k\right)\geq T\,\left(\lambda ,k\right)\\ 0,S_{r}\,\left(\lambda ,k\right)<T\,\left(\lambda ,k\right) \end{array}\right.$$

where *T*(λ,*k*) is the threshold that is dynamically updated according to the estimated SNR of the previous frame. It is worth mentioning that this threshold is set at a constant level in the literature, and we found from our pilot data that dynamic thresholds performed better than constants during our assessment, so we decided to use dynamic thresholds.

This speech-presence probability *I*(λ,*k*) can be smoothed as follows:

(6) K⁢(λ,k)=α⁢K⁢(λ,k)+(1-α)⁢I⁢(λ,k)

where *K*(λ,*k*) is the smoothed speech-presence probability, and α is a smoothing constant. The smoothing factor to be used for noise estimation can be updated using the above calculated speech-presence probability:

(7) $$\alpha_{s}\,\left(\lambda ,k\right)=\alpha_{d}+\left(1-\alpha_{d}\right)\,K\,\left(\lambda ,k\right)$$

where α_*s*_ is the smoothing factor to be used for noise estimation, and α_*d*_ is a constant. Finally, the noise power of each channel is estimated as follows:

(8) $$D\,\left(\lambda ,k\right)=\alpha_{s}\,\left(\lambda ,k\right)\,D\,\left(\lambda -1,k\right)+\left(1-\alpha_{s}\,\left(\lambda ,k\right)\right)\,{\left|Y\,\left(\lambda ,k\right)\right|}^{2}$$

### Gain Function for Noise Reduction

Using the estimated noise power, the SNR can be estimated according to

(9) $$S\,N\,R\,\left(\lambda ,k\right)=\delta \,S\,N\,R\,\left(\lambda -1,k\right)+\left(1-\delta \right)\,\left(\frac{P\,\left(\lambda ,k\right)}{D\,\left(\lambda ,k\right)}-1\right)$$

Then, we use a gain function like:

(10) $$G\,\left(\lambda ,k\right)=\frac{S\,N\,R\,\left(\lambda ,k\right)}{S\,N\,R\,\left(\lambda ,k\right)+1}$$

To suppress the noise to the maximum extent, the gain can be further adjusted:

(11) $$G_{0}\,\left(\lambda ,k\right)=\left\{ \begin{array}{c} g\;,G\left(\lambda ,k\right)<T_{g}\\ G\,\left(\lambda ,k\right),G\,\left(\lambda ,k\right)\geq T_{g} \end{array}\right.$$

where *g* is a minor constant value, and *T*_*g*_ is a dynamic threshold determined by SNR. *T*_*g*_ is also one of the key factors that determine algorithm sensitivity.

Finally, the signal power after noise reduction is as follows:

(12) $$S\,\left(\lambda ,k\right)=G_{0}\,\left(\lambda ,k\right)\,P\,\left(\lambda ,k\right)$$

### Example

**FIGURE 2:**
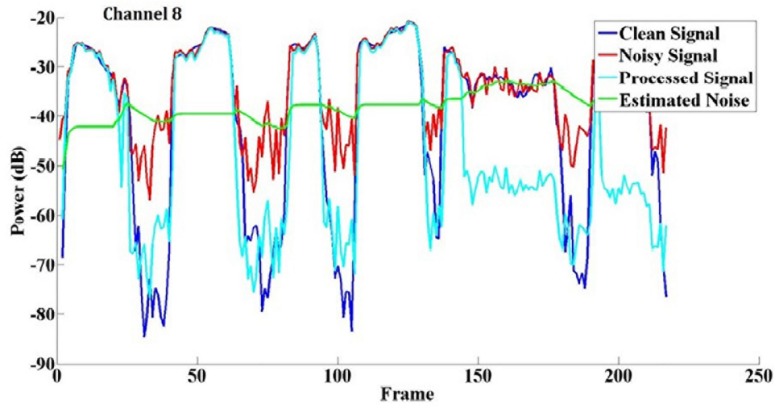
An example of noise reduction at channel 8 in SSN at +5 dB SNR. The frame shift is 8 ms.

An example of eVoice working in a speech-shaped noise (SSN) at +5 dB SNR is shown in Figure 2. eVoice was implemented with the APS coding strategy with a channel selection of 8-of-24 at a sampling rate of 16,000 Hz. Figure 2 shows the power comparison in the eighth channel, including the signals for clean speech, noisy speech, processed speech, and estimated noise plotted in different colors.

## Experiment 1: Subjective Preference and Speech Recognition in Noise

This experiment was designed to evaluate speech intelligibility with eVoice (denoted by “NR1”) compared with another NRA (denoted by “NR2”) that used a binary masking for noise reduction, as well as the APS strategy with no NRA (denoted by “APS”). NR2 uses the same noise estimation method with NR1 as described in *Noise Estimation*. After noise estimation, NR2 calculates an SNR that is used to set the gain. That is, if the SNR is higher than a threshold, set the gain to 1 (speech dominant), or a small constant if lower (noise dominant). NR2 was selected for comparison because it is as computationally effective as eVoice and the method of ideal binary masking had been studied in other CI systems (Mauger et al., 2012b). Speech intelligibility was measured with a speech-in-noise recognition test and a subjective rating questionnaire.

### Methods

#### Participants

This experiment involved 11 experienced CI users (six females and five males), aged from 20 to 59 years (mean age = 41.2 years). All were postlingually deafened adults unilaterally implanted with a CS-10A implant and using a Venus^TM^ sound processor (i.e., first generation) programed with the APS strategy. The Enduro^TM^ sound processor was fitted instead of the Venus^TM^ in this experiment. There is an option in a remote control to select whether to use an NRA (one with NR1-eVoice and the other one with NR2-Binary Masking). Demographics for individual participants are presented in Table 1. All participants’ native language was Mandarin Chinese, and participants were paid for their time and traveling expenses. Written informed consent was obtained before the experiment, and all procedures were approved by the local institution’s ethical review board.

**TABLE 1:** Demographic details of participants in experiment 1.

	**Age range**		**Profound deafness duration**	**Implanted**	**CI experience**
**Participant**	**at testing**	**Etiology**	**at implanted side (years)**	**side**	**(years)**
N1	45–49	Sudden deafness	10	L	2
N2	25–29	Unknown	2	R	2
N3	20–24	Drug induced	17	R	4
N4	45–49	Unknown	4	R	2
N5	45–49	Drug induced	35	R	2
N6	45–49	Drug induced	28	R	4
N7	35–39	Drug induced	15	L	6
N8	40–44	Sudden deafness	0.25	L	1.5
N9	55–59	Sudden deafness	4	L	3
N10	40–45	Sudden deafness	8	R	6
N11	40–45	Drug induced	17	R	6

*Abbreviations: CI, cochlear implant; F, female; L, left; M, male; R, right.*

#### Procedures and Materials

In this experiment, NR1 and NR2 performances were assessed first in a subjective evaluation, followed by a speech-in-noise recognition test.

The subjective evaluation lasted for 2 weeks. At the beginning of week 1, participants were fitted with an Enduro^TM^ processor that was incorporated with the NR1 and were asked to have a take-home trial for 1 week. During that week, participants were free to turn the NR1 on and off and use it in various everyday listening scenarios. At the end of week 1, subjective ratings were collected using the questionnaire shown in Table 2. Similar procedures were followed for the NR2 in week 2. The questionnaire consists of eight questions that cover various everyday listening scenarios. A 5-point rating scale was used to collect participants’ subjective ratings of the NR1 or NR2 in each listening scenario after each 1-week take-home use: 2, strongly agree; 1, agree; 0, neutral; -1, disagree; -2, strongly disagree.

**TABLE 2:** Questionnaire used for subjective evaluation.

Q1	The NRA has no effect on one-on-one conversations in quiet rooms
Q2	The NRA helps during multitalker (at least three talkers) conversations in quiet rooms
Q3	The NRA helps during one-on-one conversations in restaurants
Q4	The NRA noticeably suppresses noise or helps to converse with others when vehicles pass
Q5	The NRA helps during one-on-one conversations or yields clearer station announcements inside a crowded bus
Q6	The NRA helps during one-on-one conversations or provides clearer radio sound inside a car
Q7	The NRA helps during one-on-one conversations near an air conditioner or fan
Q8	The NRA helps during one-on-one conversations in a busy supermarket

*NRA refers to the noise-reduction algorithm to be evaluated (i.e., NR1 in week 1 or NR2 in week 2).*

In the test of speech recognition in noise, we used two noise types (an SSN and a babble noise) at three SNRs (5, 10, and 15 dB) to compare the three algorithms (APS, NR1, and NR2). This yielded a total of 21 test blocks (two noise types × three SNRs × three algorithms + baselines of the three algorithms in quiet). The three baseline blocks (three algorithms in quiet) were conducted first in a random order, followed by the remaining 18 blocks in a random order. We used sentence materials from two published Mandarin speech databases: the PLA General Hospital sentence recognition test (Xi et al., 2012) corpus and the House Research Institute sentence recognition test (Fu et al., 2011) corpus. The PLA General Hospital corpus consists of 12 lists each with 11 sentences, and each sentence includes six to eight key words. The House Research Institute corpus comprises 10 lists each with 10 phonetically balanced sentences, and each sentence contains seven words. All sentences were read by female speakers. Eleven of the 12 lists in the 301 corpus and all lists in the House corpus were used.

Because of the limited number of material lists, different lists from the PLA General Hospital and House Research Institute corpora were randomly assigned to blocks for each participant, with one list for each block. Special care was taken to ensure that the blocks of each algorithm used lists from the same corpus. In each block, sentences were presented in a random order, and a percentage word correctness score was calculated. Stimuli were presented in a soundproof room by a speaker located 1 m in front of the participant at a comfortable level (approximately 65 dBA). The tests were administered using QuickSTAR4TR software developed by Qianjie Fu (Emily Fu Foundation, 2019).

#### Statistical Analysis

Repeated-measures one-way analysis of variance (ANOVA) was used to analyze speech recognition in quiet. Repeated-measures three-way ANOVAs were performed to assess speech recognition in noise. Bonferroni adjustments were used for multiple comparisons.

### Results

#### Subjective Evaluation Test

**FIGURE 3:**
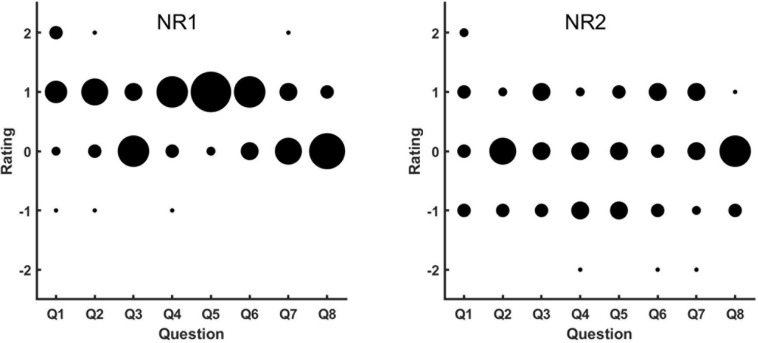
Results of subjective evaluations of NR1 **(left panel)** and NR2 **(right panel)**. The abscissa lists all eight questions used for the subjective evaluation, and the ordinate is the rating given by the participants. Along the ordinate, “-2” represents strong disagreement on the question, and “2” represents strong agreement. The larger the number, the more positive the subjective evaluation is that the NR could help in different noisy scenarios and did not impact listening in quiet settings. The size of the circles represents the number of participants who gave the corresponding ratings, with larger circles indicating more participants.

Figure 3 shows the results of the subjective ratings for NR1 and NR2.

For NR1 (i.e., eVoice), there were many positive ratings and few negative ones. Most participants gave positive ratings to Q2, Q4, Q5, and Q6, which indicated better listening experience with NR1 on than off in scenarios such as multitalker communication, at an intersection, and in a vehicle. For Q3, Q7, and Q8, most participants had neutral ratings, which corresponded to scenarios such as in a restaurant or supermarket and near an air conditioner or fan. This result suggested comparable performance between NR1 on and off in these settings. There were a few participants who give positive ratings to Q3, Q7, and Q8 (better experience with NR1 turned on in listening scenarios such as a one-on-one conversation in a restaurant, by an air conditioner or fan, or in a busy supermarket). For listening in quiet, most participants reported that NR1 had no effect on a one-on-one conversation in quiet and gave positive ratings to Q1 (the NRA had no effect on one-on-one conversations in quiet rooms).

For NR2 (i.e., binary masking), the feedback was more variable. In general, ratings were almost evenly distributed between negative and positive for all eight questions except Q2 and Q8, which means that there were participants who thought NR2 was helpful in most listening scenarios. However, comparable numbers of participants thought it was not helpful or were neutral. For Q2 and Q8, most participants gave neutral ratings, which indicate that most thought the NR2 had no effect for multitalker communication in quiet or a one-on-one conversation in a supermarket.

#### Speech Intelligibility Test

**FIGURE 4:**
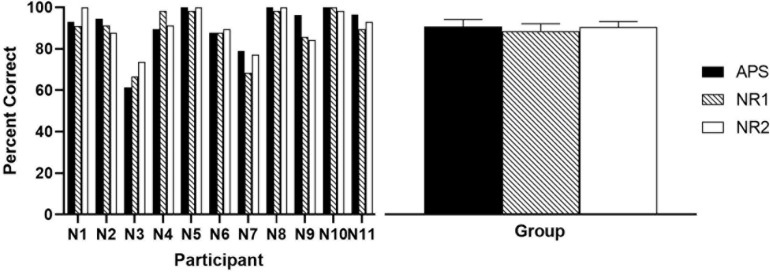
Results of speech recognition score in quiet settings. The **left panel** shows the individual percent correct scores, and the **right panel** shows the group means, with error bars indicating the standard error of group means.

Results of speech recognition in quiet are shown in Figure 4. A repeated-measures one-way ANOVA revealed no significant difference among the mean results (∼90%) of the three algorithms (*p* = 0.452).

**FIGURE 5:**
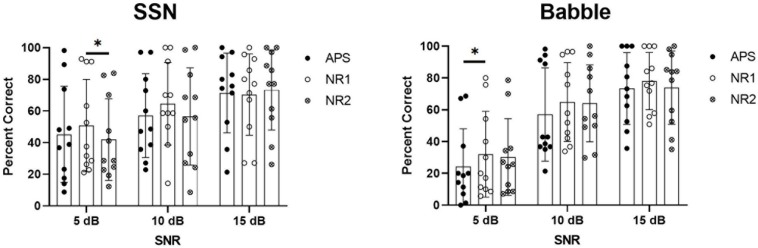
Results of speech recognition score in SSN **(left panel)** and babble noise **(right panel)**. Results of each individual participant are plotted, and the bars show the mean values, with error bars indicating the standard deviations.

Figure 5 shows the results of speech recognition in the SSN and babble noise. Statistical significance was determined using ANOVA with the percent correct scores as the dependent variable and the noise type (SSN or babble), SNR (5, 10, or 15 dB), and algorithm (APS, NR1, or NR2) as within-subject factors. Tests of within-subjects effects indicated a significant effect of noise type (*p* = 0.022), SNR (*p* < 0.001), and algorithm (*p* = 0.002), as well as significant interactions between noise type and SNR (*p* < 0.001). Pairwise comparisons revealed that the overall performance of NR1 was significantly better than APS (*p* = 0.001) and NR2 (*p* = 0.016), and there was no significant difference between APS and NR2 (*p* = 0.612). When noise type and SNR were fixed to determine the effect of algorithms at specific SNRs in a particular noise type, NR1 performed significantly better than NR2 at the 5-dB SNR in the SSN (*p* = 0.010) and also significantly better than APS (*p* = 0.027) at the 5-dB SNR in the babble noise. In both the SSN and babble noise at the SNRs of 10 and 15 dB, there were no significant differences among the three algorithms. However, higher mean scores of NR1 could be observed against APS and NR2 at the 10-dB SNR in SSN (nearly eight percentage points), as well as at the 10-dB SNR (eight percentage points higher than APS) and 15-dB SNR (∼5 percentage points higher than APS and NR2) in the babble noise, although these improvements were not statistically significant.

### Short Summary

In this experiment, we tested two NRAs: eVoice (NR1) and another that used binary masking (NR2). Both use the same noise estimation process but differ in the noise cancelation process. NR1 uses a smoothing gain function, whereas NR2 uses a binary masking. The subjective evaluation ratings show that NR1 was positively reviewed, whereas ratings of NR2 were almost evenly distributed from negative to positive, with a slight dominance of neutral responses. The speech recognition test results indicate overall better performance of NR1 compared to NR2 and APS. However, a significant benefit was only found at 5-dB SNR. The above results demonstrate that NR1 had better performance than NR2 for both speech recognition tests and subjective evaluations.

## Experiment 2: Speech Reception Threshold Test

### Rationale

The hypothesized significant benefit of eVoice was not always supported by the results of the first experiment. One reason may be from the fixed SNR procedure and large performance variance in the cohort. From the results of Experiment 1 (left panel in Figure 5), we noticed that the ceiling effect could be observed in some participants at the SNR of 15 dB, and the floor effect could be observed at the SNR of 5 dB. Speech perception in noise varied dramatically among participants, even at the same SNR in the same noise. This indicates a limit of testing percent correct scores at fixed SNRs because this type of test is not able to exclude potential ceiling and floor effects. To overcome this limitation, we designed Experiment 2, which used an adaptive speech reception threshold (SRT) test to measure the potential benefits of eVoice.

In the first experiment, we found clearly that NR1 (i.e., eVoice) provided better performance than NR2 (i.e., the ideal binary one) in the subjective test, although little improvement was observed in the speech-in-noise recognition test. To further explore the potential of eVoice and to save experiment time, only NR1 was evaluated in the second experiment.

### Methods

#### Participants

Eight experienced CI users were recruited for this experiment (five females and three males, aged from 23 to 62 years with a mean of 43.6 years). All spoke Mandarin Chinese as their native language. They were all postlingually deafened adults unilaterally implanted with a CS-10A implant and used Enduro^TM^ devices as their clinical processors, programed with the APS coding strategy with a remote control option to switch eVoice on or off. Demographic data for individual participants are presented in Table 3. Participants were compensated for their time and traveling expenses. All provided informed consent before the experiment, and all procedures were approved by the local institution’s ethical review board.

**TABLE 3:** Demographic details of participants in experiment 2.

	**Age range**		**Profound deafness duration**	**Implanted**	**CI experience**	**Enduro**
**Participant**	**at testing**	**Etiology**	**at implanted side (years)**	**side**	**(years)**	**experience (years)**
N12	20–24	Drug induced	1	R	6	1
N13	50–54	Unknown	0.4	L	6	0.5
N14	30–34	Sudden deafness	1	R	1.5	1
N15	45–49	Unknown	5	L	7	3
N16	50–54	LAVS	1	L	6	1
N9	60–64	Sudden deafness	4	L	6	3
N10	45–49	Sudden deafness	8	R	9	3
N17	35–39	Unknown	7	L	8	0.5

*Abbreviations: CI, cochlear implant; F, female; L, left; LAVS, large vestibular aqueduct syndrome; M, male; R, right.*

#### Materials and Procedures

An adaptive staircase SRT in noise test was administered to further evaluate the performance of eVoice. This SRT measurement method was adopted from our previous studies (Meng et al., 2016, 2019) with two minor changes: (1) the stimulus presentation time was reduced from three at most to two at most, and (2) the correctness judgment threshold was changed from 50% words in a sentence to 80% words. The first was done to reduce experiment time. The second was for tracking a higher threshold, which is more indicative for a true understanding. Therefore, we were actually tracking a threshold around which the subjects have a 50% chance to obtain 80% correctness.

The Mandarin Hearing in Noise Test (MHINT) corpus (Wong et al., 2007) recorded by a single male speaker was used. There are 12 lists for formal tests and 2 lists for practice, with 20 sentences in each list, and 10 words in each sentence. In this experiment, 10 of 12 formal test lists were used as target speech in the formal tests, and both practice lists were used in the training stage to familiarize participants with the test procedures.

The SRTs for each condition with and without eVoice were tested. For each condition, two types of background noise were used: SSN and babble noise, which were generated using the method described in section “Speech Stimuli and Tasks” of Experiment 2 in Meng et al. (2019). The SRT for each condition–background combination was tested twice using two different MHINT lists, and the results were averaged between the two lists as the final SRT. Speech intelligibility for each condition in quiet was also measured using one MHINT list. Therefore, a total of 10 lists were used for testing (two backgrounds × two conditions × two lists per combination + two lists for speech intelligibility in quiet). The order of lists and conditions was randomized across participants. Prior to the formal test, two practice lists were used to familiarize participants with the test procedures of the SRT and the speech intelligibility in quiet test. During the test, each sentence was presented at most twice on the request of the participants; participants were instructed to repeat words that form a sentence with a meaning, and no feedback was given.

The SNR in each trial was adapted by changing the level of target speech with fixed background noise. Participants were instructed to repeat as many words as they could, and the target level was decreased if no less than eight of the words were repeated correctly; otherwise, the target level was increased. The step size was 8 dB before the second reversal, followed by 4 dB before the fourth reversal and 2 dB for the remaining reversals. The arithmetic mean of the SNRs of the last eight sentences was calculated and recorded as the final SRT.

It is worth mentioning that the babble noise used in this study consisted of voices of the same talker as the target speech (Meng et al., 2019), which is extremely challenging for any NRA. Additional information about the procedures and materials can be obtained from Meng et al. (2016, 2019).

### Results

The eight CI users listed in Table 3 participated in this experiment, but N17 was found to have auditory neuropathy. Therefore, N17 data were excluded from the analyses.

**FIGURE 6:**
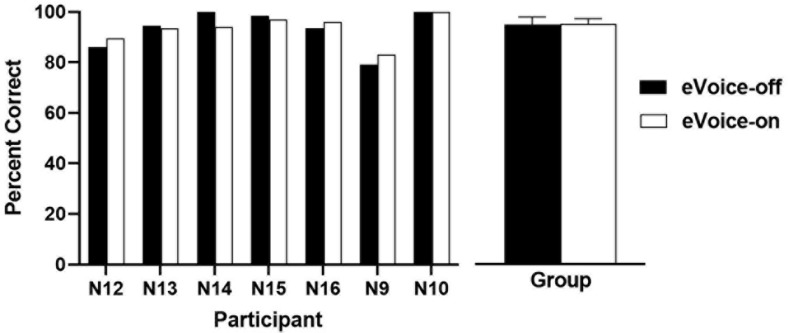
The speech recognition scores in quiet with eVoice-off and eVoice-on. The **left panel** shows the individual scores, and the **right panel** shows the group means, with error bars showing the standard errors of group means.

Results of speech recognition in quiet are shown in Figure 6. The group mean scores were 93.1 and 93.3% for eVoice-off and eVoice-on, respectively. A two-tailed paired-samples *t*-test showed no significant difference between the two conditions (*t*(6) = −0.162, *p* = 0.877).

**FIGURE 7:**
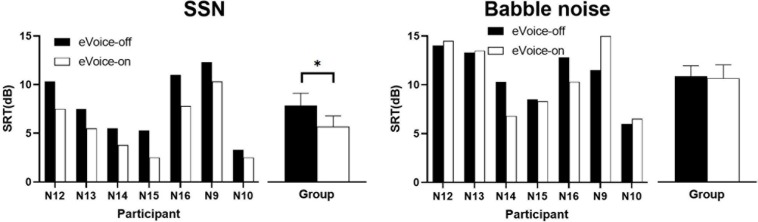
Results of SRT in the SSN **(left panel)** and babble noise **(right panel)**. Individual SRTs are shown on the left, and the group mean SRTs are shown on the right. Error bars show the standard error of group means. The significant difference is illustrated by the asterisk (*p* < 0.05).

Figure 7 shows the results of the SRTs in the SSN (left panel) and babble noise (right panel). In the SSN, every participant had lower SRTs with eVoice-on than with eVoice-off. The group mean SRTs were 7.9 and 5.7 dB for eVoice-off and eVoice-on, respectively. This 2.2-dB difference was a statistically significant improvement (*t*(6) = 6.892, *p* < 0.001).

In the babble noise, group mean SRTs of 10.9 and 10.7 dB were observed for eVoice-off and eVoice-on, respectively. A two-tailed paired-samples *t* test revealed no significant difference between the two conditions (*t*(6) = 0.249, *p* = 0.812).

### Short Summary

The aim of this experiment was to quantify the benefit introduced by eVoice for speech intelligibility and exclude potential ceiling and floor effects. Speech intelligibility was measured using an adaptive SRT test with two different backgrounds: SSN and babble noise. There was no significant difference in speech recognition rates in quiet settings. This result indicates that eVoice would not affect speech perception in quiet. eVoice yielded an SRT decrease of 2.2 dB in SSN, whereas no significant effect was found in SRTs in babble noise.

## Discussion

In this study, we examined eVoice, the first noise-suppression technique in Nurotron^®^ CIs. eVoice is a single-channel NRA implemented within the APS strategy in the Enduro processor. Two experiments were conducted to evaluate this algorithm. First, the performance of eVoice was compared with another binary-masking method in a speech recognition test and also underwent a subjective evaluation in Experiment 1 (*N* = 11). The eVoice performed slightly better than the binary-masking NRA. Then, the more indicative adaptive SRT test was conducted to quantify the noise reduction effect of eVoice on speech intelligibility in Experiment 2 (*N* = 7). Comparing eVoice on and off, there was a 2.2-dB SRT benefit in stationary noise and no difference in quiet and non-stationary noise.

Compared to other single-channel NRAs implemented in CI strategies, eVoice has comparable performance with those reported in the literature. For example, a single-channel NRA implemented in the ACE strategy was found to have an SRT benefit of up to 2.14 dB in stationary noise (Dawson et al., 2011). The ClearVoice implemented in the HiRes 120 strategy used a time window of 1.3 s for noise estimation and yielded a percent correct score increase of up to 24 percentage points (Buechner et al., 2010). This may translate to a 1.3- to 3.4-dB SRT decrease according to the literature that for typical speech materials, a 1-dB SRT decrease leads to 7- to 19-percentage-point increase in the percent correct score (Moore, 2007). However, significant benefits in non-stationary noise are seldom reported in the literature, which may indicate a limit of traditional single-channel NRAs. More advanced techniques should be developed to improve speech perception in non-stationary noise for CI users.

This article is significant from the implantees’ and the audiologists’ perspectives. For a new system with a quickly growing number of users, this report on eVoice is useful for understanding the system and the new noise reduction method. For a new NRA in CIs, two questions are of great concern to users and audiologists: (1) whether this NRA really works in various types of noises and (2) to what extent users can benefit from it. Our results demonstrate that eVoice can improve speech intelligibility in stationary noise and does not affect speech perception in quiet and non-stationary noise. This is because eVoice is a monaural SNR estimation–based algorithm that assumes that the noise is relatively stationary compared with speech. We found that some users of the Enduro processor might have not noticed the existence of this NRA, and their audiologists can advise or remind them to turn eVoice on to improve their speech perception performance in noise.

Another significant contribution of this article is to inspire people to rethink noise management for CI systems. Researchers should consider the assumptions about directionality and complex non-linear patterns that can be computationally modeled by signal processing or machine learning (e.g., Bianco et al., 2019; Gong et al., 2019). Previous studies and present work provide considerable support for optimizing and updating noise-suppression techniques to improve speech-in-noise recognition for CI users.

## Data Availability Statement

The datasets generated for this study are available on request to the corresponding author.

## Ethics Statement

The studies involving human participants were reviewed and approved by Medical Ethics Committee of Shenzhen University. The patients/participants provided their written informed consent to participate in this study.

## Author Contributions

All authors conceived and designed the analysis and wrote the manuscript. HZ and NW collected the data.

## Conflict of Interest

Nurotron provided some compensation to the subjects and accommodation to HZ during Experiment 2. NW was employed by the company Nurotron Biotechnology Inc. The remaining authors declare that the research was conducted in the absence of any commercial or financial relationships that could be construed as a potential conflict of interest.
